# Human Frataxin Folds Via an Intermediate State. Role of the C-Terminal Region

**DOI:** 10.1038/srep20782

**Published:** 2016-02-09

**Authors:** Santiago E. Faraj, Rodolfo M. González-Lebrero, Ernesto A. Roman, Javier Santos

**Affiliations:** 1 Instituto de Química y Físico-Química Biológicas, Universidad de Buenos Aires, Junín 956, 1113AAD, Buenos Aires, Argentina

## Abstract

The aim of this study is to investigate the folding reaction of human frataxin, whose deficiency causes the neurodegenerative disease Friedreich’s Ataxia (FRDA). The characterization of different conformational states would provide knowledge about how frataxin can be stabilized without altering its functionality. Wild-type human frataxin and a set of mutants, including two highly destabilized FRDA-associated variants were studied by urea-induced folding/unfolding in a rapid mixing device and followed by circular dichroism. The analysis clearly indicates the existence of an intermediate state (I) in the folding route with significant secondary structure content but relatively low compactness, compared with the native ensemble. However, at high NaCl concentrations I-state gains substantial compaction, and the unfolding barrier is strongly affected, revealing the importance of electrostatics in the folding mechanism. The role of the C-terminal region (CTR), the key determinant of frataxin stability, was also studied. Simulations consistently with experiments revealed that this stretch is essentially unstructured, in the most compact transition state ensemble (TSE2). The complete truncation of the CTR drastically destabilizes the native state without altering TSE2. Results presented here shed light on the folding mechanism of frataxin, opening the possibility of mutating it to generate hyperstable variants without altering their folding kinetics.

Frataxin (FXN) is a highly conserved protein that plays a crucial role in the iron metabolism. Several functions have been proposed for this protein, including iron chaperone activity and regulation of the iron-sulfur (Fe-S) cluster assembly. In eukaryotes, FXN promotes the transfer of –SH groups from cysteine desulfurase (ISCS) to iron-sulfur cluster assembly enzyme (ISCU), and helps in the assembly of the Fe−S cluster, which are essential prosthetic groups with a key role in many biological processes. Also, FXN knockdown causes severe alterations in the levels of Fe-S cluster-containing enzymes. This protein has a large and conserved anionic surface (the acidic ridge) that involves Glu and Asp residues from helix α1, loop1, and strand β1. FXN binds to ISCS through electrostatic interactions between the acidic ridge and a counterpart cluster of positively charged residues on the surface of ISCS^1^,^2^,^3^. This interaction may be competed by ferredoxin, which also exhibits an anionic patch^4^. In addition, FXN may interact with holo and apo ISCU^5^, and the interaction might be modulated by iron^6^ and possibly by the redox state of ISCU.

FXN is a focus of attention because its deficiency causes a neurodegenerative disease known as Friedreich’s ataxia (FRDA)^7^,^8^,^9^. Although the mechanism by which FXN deficit causes neuro- and cardio-degeneration is unclear, very interesting findings by Hayashi and coworkers suggest neuroinflammatory mechanisms in FRDA, including prostaglandin synthesis^10^.

Human frataxin (hFXN) is synthesised in the cytoplasm and imported into the mitochondria via a signal peptide. In this organelle, the N-terminal stretch is removed in two sequential steps of proteolysis. The protein is processed to yield the intermediate form hFXN42–210 and, afterwards, the mature form hFXN81-210^11^,^12^. It is worth noting that residues 81–90 likely form a disordered stretch^13^,^14^. The structure of hFXN90-210 has been resolved by NMR^15^ and crystallography^16^,^17^,^18^, showing that it is made up of an antiparallel five-stranded β-sheet, and two parallel α helices forming an α/β sandwich (Fig. 1A). hFXN is a very stable protein (∼9 kcal/mol^**−**1 ^19,20). Native state dynamics of hFXN has been deeply studied by NMR^19^,^21^ in a broad range of timescales and also by molecular dynamics simulations (MDs^19^,^20^). A number of FRDA-associated mutants were studied and characterized from the point of view of their effects on thermodynamic stability, internal dynamics and biological activity^3^,^16^,^21^,^22^.

The C-terminal region (CTR) of the eukaryotic variants is usually larger than the prokaryotic ones, being this fact a key topological difference between FXNs. One exception is yeast FXN (yFXN), which lacks CTR^23^. This fact opens question about the structural role of this intriguing and non-conserved region of the protein. In particular, we have focused on mutations located in the CTR. We have observed that deletion of the CTR produces a complete alteration of hFXN internal dynamics and yields a critical destabilization of the protein (Δ*G*°_NU_ = 1.0 kcal mol^**−**1^ for the truncated variant^19^,^20^), suggesting why the closely related FRDA-associated mutant hFXN81-193 causes the disease^24^. In the case of hFXN90-195, ∼10% of the molecules are in the unfolded state at room temperature^20^. Moreover, we studied in detail the perturbations produced by the FRDA-related mutation L198R^25^. We observed that this mutation locally alters internal motions, mainly involving residues that are in close contact with L198 in wild-type hFXN. In addition, this mutation produces a large effect on global stability of the protein and significantly reduces its iron binding capability, an activity that may be related to FXN function^19^.

The folding mechanism of the monomeric form of yeast variant has been investigated and, in principle, a two-state model is sufficient to describe its behavior. Based on the curvature of chevron plots of yFXN, it has been suggested that its folding involves a broad smooth free energy barrier^26^,^27^. The presence of curvatures in the arms of chevron plots has been observed previously in many other proteins and associated to the existence of high-energy intermediate states^28^,^29^,^30^,^31^,^32^.

Using NMR techniques, Vilanova and coworkers demonstrated that the folded form of yFXN and a folding intermediate are in slow exchange at equilibrium at room temperature^33^. The protein is destabilized by localized energetic frustration arising from acidic residues (in strands β1 and β2). This region of the protein is locally unfolded in the intermediate, as judged by the analysis of backbone chemical shifts. Equilibrium, however, may be shifted to the folded form in the presence of low salt concentrations.

On the other hand, the folding mechanism of hFXN has yet to be studied carefully. It is important to understand this mechanism in detail, ultimately to learn how to stabilize the functional form of the protein, to enhance its intra mitochondrial concentration in the cells of FRDA patients. This has high biological relevance given that the existence of partially folded states and slow folding reactions may regulate protein translocation processes across the mitochondrial membranes^34^,^35^.

In this study we have researched the folding mechanism of hFXN by means of urea-induced unfolding/refolding experiments followed by circular dichroism (CD). Rapid kinetic measurements allowed us to detect an intermediate state: U↔I↔N, indicating a rough folding landscape for this protein. The picture that emerges from hFXN folding experiments is considerably more complex than what was recently described for the yFXN homolog, and sets a new framework for hFXN, and perhaps for the CyaY protein family as well.

## Results

### The Conformation and Stability of hFXN Variants

The presence of the CTR makes hFXN topologically different from yFXN, which is devoid of this stretch. In addition, FXN from *E. coli* (ecFXN) has a shorter CTR compared with the human variant. Interestingly, both the extension and biochemical properties of this region were previously correlated with differences in the thermodynamic stability of these variants, showing that the stability of hFXN>ecFXN>yFXN^23^. Thus, it is relevant to investigate the involvement of this part of the protein in the folding mechanism given the fact that the CTR is a critical determinant of stability.

**Figure 1:**
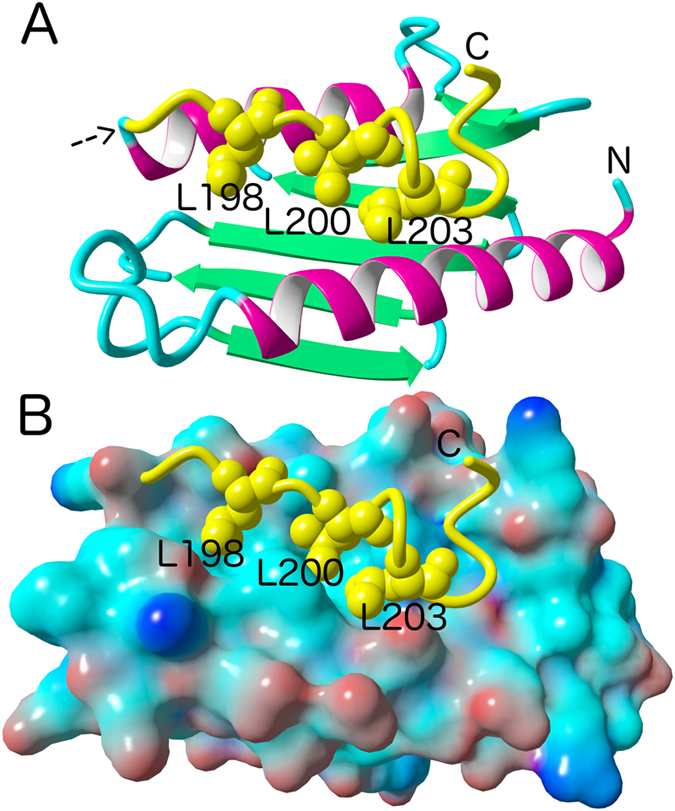
The hFXN Structure. (**A**) Ribbon representation of hFXN. (**B**) A representation of the molecular surface corresponding to residues 90-195 is shown, the CTR is represented by a yellow ribbon (residues 195-208) and residues that were mutated in this study (L198, L200 and L203) are represented in van der Waals in yellow. The model was generated with YASARA view software and the PDB ID: 1EKG. The arrow in A indicates the site in the backbone where CTR starts.

**Figure 2:**
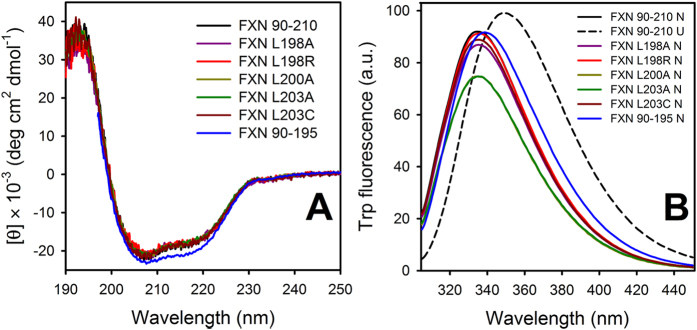
Spectroscopic Characterization of hFXN Variants. Far-UV CD **(A)** and Trp fluorescence spectra **(B)** of hFXN variants are shown. Fluorescence spectra corresponding to the urea-induced unfolded state for wild-type hFXN are also shown (dashed line). Buffer was 20 mM Tris-HCl, 100 mM NaCl, pH 7.0. Unfolding was performed by the addition of 7.0 M urea and incubation for at least 3 h. Protein concentration was 10 μM.

More specifically, we focused on residues L198, L200 and L203 that conform a cluster of hydrophobic interactions on one side of the CTR (Fig. 1B). In this context, we studied folding dynamics of wild-type hFXN and five CTR point mutants: L198R, L198A, L200A, L203A and L203C (Fig. 1). Moreover, we studied how the complete deletion of the CTR affects folding kinetics. In Fig. 2, far-UV CD and Trp fluorescence spectra corresponding to hFXN variants are shown.

Under native conditions, all variants exhibit native-like features, compatible with the folded state of hFXN. Although they have a similar secondary structure content, as judged by CD bands and λ_MAX_ of emission, mutants L200A and L203A exhibit a slight quenching of Trp fluorescence intensity, most likely due to subtle packing rearrangement. The stability of the hFXN variants was evaluated at 25° C by urea-induced unfolding experiments under equilibrium conditions. The set of full-length variants under study covers a broad range of thermodynamic stabilities. Most of them are significantly destabilized compared to the wild-type protein (Table 1, right). The exception is L203C, which is ∼1.0 kcal mol^**−**1^ more stable than the wild-type variant^19^ (Table 1). Most of the variants show a completely reversible and cooperative folding/unfolding process, and protein aggregation is not observed under the full-range of studied urea concentrations or buffer conditions. However, L203A exhibits high aggregation during refolding by dilution. Unfortunately, this behavior precludes further experimental study of this mutant.

### A Three-State Model Properly Describes the hFXN Folding Mechanism

**Figure 3:**
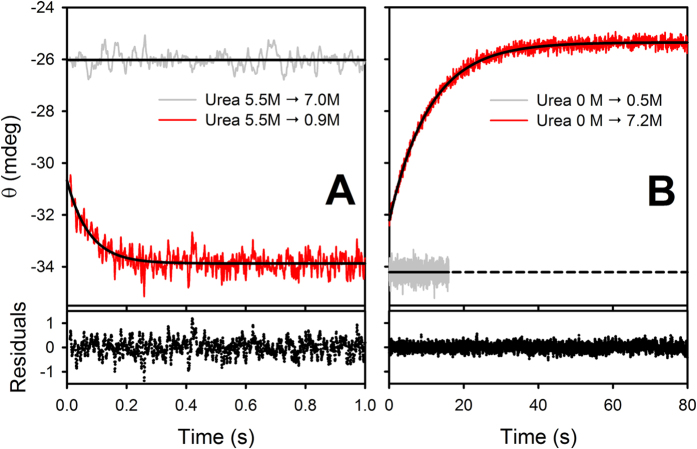
Time Traces. Folding **(A)** and unfolding **(B)** of wild-type hFXN followed by far-UV CD (red). Baselines corresponding to the rapid mixing of unfolded protein and urea solution (gray, panel **A**) or native protein with buffer without urea (gray, panel **B**) are shown. Buffer was 20 mM sodium phosphate, 100 mM NaCl, pH 7.0. The continuous lines are plots of a single exponential function of time plus a time-independent term (**Equation** 1) for the values of the parameters that gave best fit to the experimental data. The differences between the experimental data and the fitted function are shown below.

We studied the hFXN folding mechanism by rapid mixing experiments. In Fig. 3, we show representative time traces corresponding to the folding and unfolding reactions of the wild-type protein followed by the change in the CD ellipticity signal.

Good descriptions of the time courses were obtained by fitting a single exponential function of time plus a time-independent term **(Equation** 1). Moreover, according to the *Akaike Information Criterion* (AIC, **Equation** 5), the goodness of fit did not improve by using a sum of two exponential functions of time.


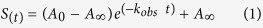


**Figure 4:**
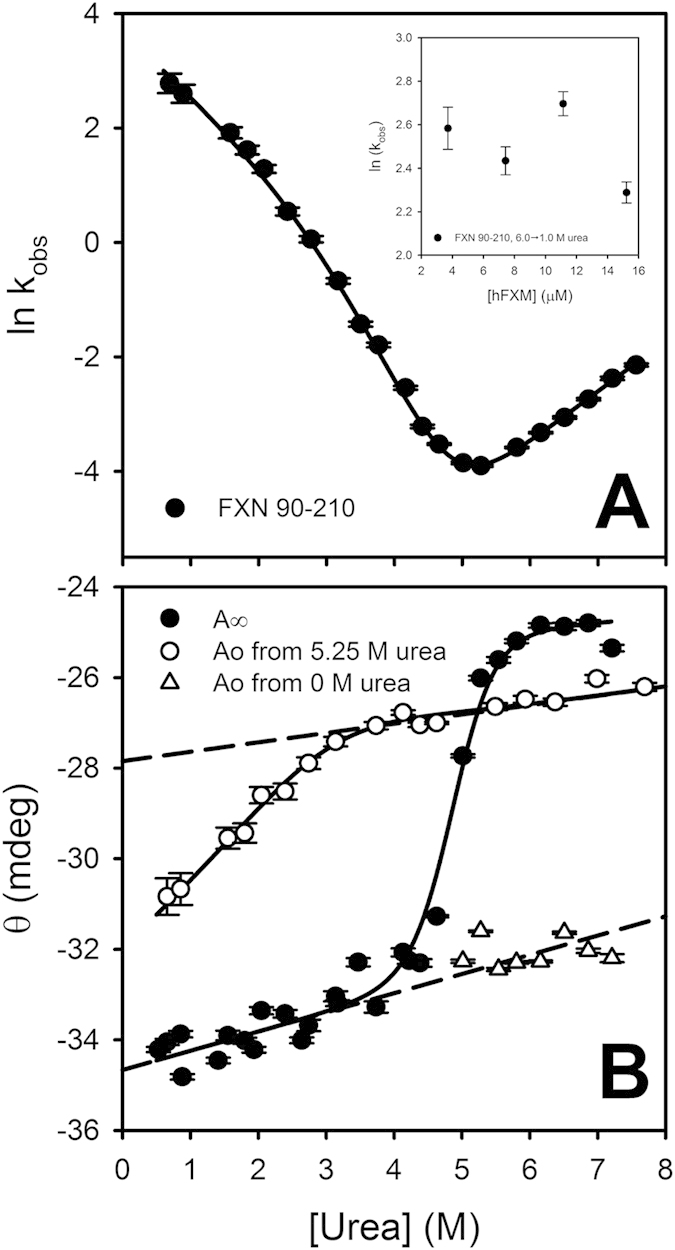
The dependence on urea concentration of k_obs_, *A*_0_ and *A*_∞_ that gave best fit to the experimental data. (Panel **A**) is a plot of k_obs_. (Panel **B**) is a plot of *A*_∞_ (signal at t → ∞, filled circles) and *A*_0_ (signal at t = 0), which were obtained from unfolding and refolding experiments (open triangles and circles, respectively). Buffer was 20 mM sodium phosphate, 100mM NaCl, pH 7.0. Prior to the initiation of the refolding reaction, the protein was incubated in a buffer with 5.25 M urea. The inset in panel A shows the best fitting values of k_obs_ obtained from refolding experiments at 1 M urea (final concentration) with different protein concentration. The continuous lines are the plot of equations derived from the three-state model, (Eqs 2), with the best fitting values of the parameters that gave best fit to k_obs_, A_∞_ and A_0_ respectively. The dashed lines represent the change in the spectroscopic signal with urea concentration, according to the fractions of native, intermediate and unfolded states at 5.25 M urea. Vertical bars represent the experimental errors.

In **Equation** 1, *A*_∞_ and *A*_0_ are the signals when t tends to ∞ or at t = 0, respectively, and k_obs_ is the rate coefficient (the observed constant). The best fitting values of k_obs_, *A*_0_ and *A*_∞_ are plotted in Fig. 4.

It is worthy of note that far-UV CD signals, at the end of the reactions (*A*_∞_, Fig. 4), parallel the equilibrium-unfolding curve obtained in independent experiments (Table 1, Cm values). Thus, folding and unfolding kinetics were followed until equilibrium.

A rollover is observed in the refolding branch of the chevron plot of hFXN (left, low urea concentrations) (Fig. 4A). This kind of behavior has been previously linked to protein association and aggregation^36^, prolyl isomerization^37^,^38^, or the existence of at least one intermediate state^28^,^29^,^30^,^39^,^40^. In addition, the curvature of chevron plots has also been associated to the effect of denaturant agents on the transition state ensemble (TSE), which produce a movement of the speed-limiting barrier (the so-called “broad transition barrier” in a two-state folding reaction)^41^. First of all, we ruled out hFXN aggregation under these experimental conditions because no changes in the k_obs_ were detected when protein concentration was varied between 3 and 15 μM (Fig. 4A**, inset**). In addition, even at high protein concentrations, all variants are completely monomeric, as judged by multi-angle light scattering experiments (data not shown). On the other hand, we ruled out peptidyl-prolyl cis/trans isomerization influences due to the absence of an extra phase, as observed in other proteins and peptide models^42^,^43^ (slow processes with *k*_iso_ coefficient that do not vary with denaturant concentration).

In the first milliseconds of the refolding reaction, there is a gain in the CD signal, which is compatible with a fast increase in the secondary structure content. At low urea concentrations, the signals at the beginning of the recorded refolding kinetics (t = 0) do not match the extrapolation of the unfolded baseline obtained by dilution of unfolded protein in urea (Fig. 4B), indicating that there is a significant change in the signal during the dead time (10 ms) of the refolding reaction. The presence of this burst phase is compatible with at least one intermediate state (I) in the folding reaction (see below).

Thus, even though it has been suggested that curvatures of chevron plots might have their origin in the alteration of the TSE with denaturant (e.g., a change in compactness), as in the case of the yFXN homolog, the applicability of a two-state model seems to be unrealistic in the case of hFXN because of the presence of the burst phase. Altogether, these results firmly indicate that the hFXN folding mechanism can be explained by the following three-state model:


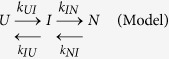


in which U is unfolded, I is the intermediate and N is the native state ensemble, and k_UI_, k_IU_, k_IN_ and k_NI_ are the rate constants.

The analytic solution of the equation derived from the three-state model is the sum of two exponential functions of time, each one characterized by an apparent rate coefficient. In the case of hFXN, we observed a single exponential phase. However, we also detected a change in the signal during the dead time of the refolding reaction, which can be explained considering that k_UI_ is significantly higher than the rate constant k_IN_. Given that we cannot obtain reliable kinetic information on k_UI_ and k_IU_, we decided to consider rapid equilibrium between U and I states, characterized by the equilibrium constant *K*_IU_ (the ratio of the rate constants k_UI_/k_IU_).

Under rapid equilibrium, the analytic solution of the model is a single exponential function of time, in which the signal (S) at a certain time (t) is defined by **Equation** 1, whereas the coefficient k_obs_, the signal at equilibrium (*A*_∞_) and the signal at t=0 (*A*_0_) are defined by **Equations** 2, respectively:


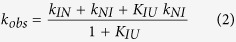



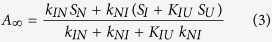



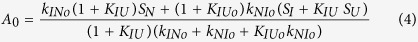


where S_U_, S_I_ and S_N_ are the signals of the unfolded, intermediate and native states, respectively, and k_INo_ and k_NIo_, and *K*_IUo_ are the rate constants and the equilibrium constant before mixing and changing the denaturant concentration.

**Equations** 2 were simultaneously fitted to k_obs_, *A*_∞_ and *A*_0_ values. As can be seen in Fig. 4, the three-state model properly describes the dependence of these parameters on urea concentration. The best fitting values of rate and equilibrium constants of the model and m_i_ parameters are shown in Table 2.

Although we were unable to obtain values for k_UI_ and k_IU_, because information of the transition state ensemble between U and I (TSE1) was lost, we were able to determine *K*_IU_, k_IN_ and k_NI_ and their dependences on urea concentration. This enabled partial characterization of the I state (accessible surface area and compaction) and full characterization of the transition state ensemble between I and N states (TSE2) in terms of its energetics relative to the ground states. It was possible to analyse progression of the folding reaction and the acquisition of structure in terms of the α_D_ parameter^44^, (α_urea_ in our case, **Equations** 8 and 9) that reflects the relative sensitivity of the intermediate and transition state ensembles to changes in denaturant concentration. The parameter α_urea_ is interpreted as the amount of accessible surface area of the transition states (or other ensemble) relative to the reference states^44^. While U and N states are characterized by α_urea_ values of 0 and 1, respectively, I and TSE2 exhibit α_urea_ values of 0.44 ± 0.01 and 0.72 ± 0.01, correspondingly. This result indicates that TSE2 is considerably more compact than I.

**Figure 5:**
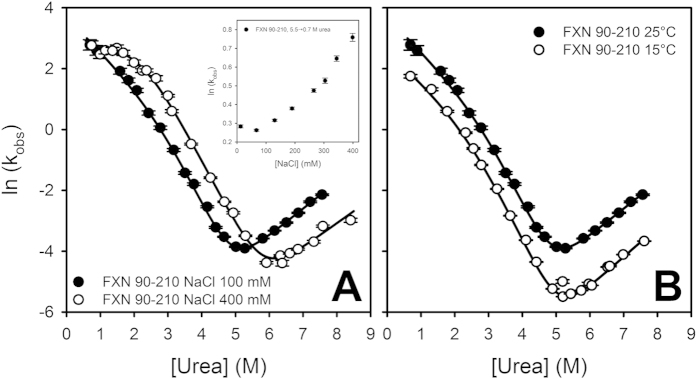
Chevron Plots at different NaCl concentrations **(A).** 100 mM (filled symbols) and 400 mM NaCl (open symbols). The variation of k_obs_ with NaCl concentration (from 0 to 400 mM) is shown in the inset; in this experiment initial and final urea concentration was concentration was 5.5 and 0.7 M, respectively. **(B)** Chevron plots at 25 °C and 15 °C. The continuous lines are the plot of Equation 2 with the best fitting values of the parameters that gave best fit. Vertical bars represent the experimental errors. Buffer was 20 mM sodium phosphate, 100 mM NaCl, pH 7.0.

Because sodium chloride has a significant effect on the stability of hFXN^20^,^23^ and FXNs from other organisms^33^,^45^, folding/unfolding kinetics was also studied in the presence of a higher concentration of NaCl (Fig. 5A).

The general profile of folding/unfolding for hFXN at 400 mM is similar to the profile observed at 100 mM and the three-state model also provides a good description of the experimental results. Some peculiarities are evident: (a) the rate constant *k*_NI_ shifts from (1.4 ± 0.1) 10^**−**4^ s^**−**1^ (100 mM) to (8.1 ± 1.2) 10^**−**5^ s^**−**1^ (400 mM), accounting for an increase in the activation barrier (N → TSE2), which indicates that NaCl stabilizes N with respect to the more expanded ensemble TSE2; (b) m_IN_, the dependence on denaturant concentration of the constant rate k_IN_, is significantly lower when NaCl concentration is higher, suggesting that the transition I → TSE2 involves a smaller change in surface area at 400 mM (α_urea I_ = 0.71 ± 0.02) than at 100 mM NaCl, and most likely, at higher salt concentrations the compaction of the I state increases, whereas the compaction of TSE2 remains almost unaltered (α_urea TSE2_= 0.73 ± 0.01 at 400 mM NaCl); (c) the equilibrium constant *K*_IU_ decreases ∼3 times when NaCl concentration is increased from 100 to 400 mM (0.033 ± 0.007 and 0.011 ± 0.001, respectively) suggesting the stabilization of I state with respect to U; (d) a significant increase in stability occurs: ΔΔ*G*°_NU_ = 0.6 kcal mol^**−**1^; (e) most likely, there is a slight compaction (∼10%) of the U state, as judged by a decrease in the m_NU_ value (1.68 ± 0.02 kcal mol^**−**1^ M^**−**1^) at a higher NaCl concentration.

In addition, we evaluated wild-type hFXN folding/unfolding kinetics at 15 °C (Fig. 5B) and found that the three-state model explains the results obtained, indicating that there are no significant changes in mechanism in this temperature range (compare rate constants and their dependences on denaturant in Tables 2 and 3).

### The CTR is involved in the stabilization of the native state, but it does not play a role in the energetics of either the TSE or the intermediate state

**Figure 6:**
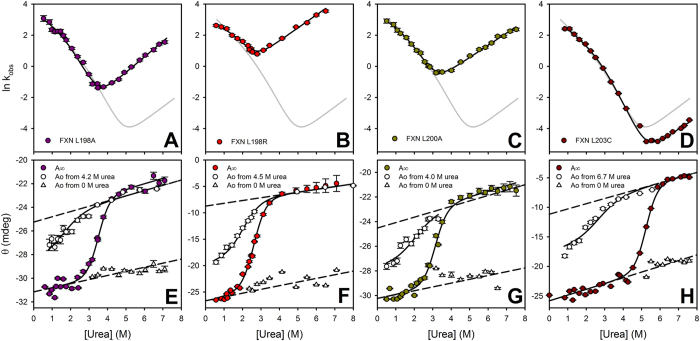
The dependence on urea concentration of k_obs_, *A*_0_ and *A*_∞_ that gave best fit to the experimental data for hFXN variants L198A (**A**,**E**), L198R (**B**,**F**), L200A (**C,G**), and L203C (**D,H**). (Panels **A–D**) are plots of k_obs_. (Panels **E–H**) are plots of *A*_∞_ (signal at t → ∞, filled circles) and *A*_0_ (signal at t = 0), which were obtained from unfolding and refolding experiments (open triangles and circles, respectively). Buffer was 20 mM sodium phosphate, 100 mM NaCl, pH 7.0. In the refolding experiments, proteins were incubated in a buffer with urea (L198A: 4.2 M, L198R: 4.5 M, L200A: 4.0 M and L203C: 6.7 M) prior to initiation of the refolding reaction. The dashed lines represent the change in the spectroscopic signal for each variant with urea concentration, according to the fractions of native, intermediate and unfolded states prior to the initiation of the refolding reaction. The continuous lines are the plot of equations derived from the three-state model, (Eqs 2), with the best fitting values of the parameters that gave best fit to k_obs_, *A*_∞_ and *A*_0_ respectively. Vertical bars represent experimental errors. Gray curves in upper panels are the plot derived from the three-state model for the wild-type variant (Figure 4A).

As for wild-type hFXN, a single exponential function of time was enough to properly describe the time traces for all full-length point mutants. In addition, all mutants show a burst phase in the refolding branch—confirming the existence of the intermediate state I—and a rollover in the left arm of the chevron plot, similar to that observed for the wild-type protein (Fig. 6). Based on these observations, we analyzed the results using the three-state model used for the wild-type protein.

No significant differences were found for the dependences of rate constants (Table 2) on denaturant concentration, which suggests that the position of each ensemble along the reaction coordinate (the compaction) is the same for all variants. On the other hand, k_NI_ values notably vary (four orders of magnitude, Table 2), whereas the other constants k_IN_, and *K*_IU_ do not significantly change. Moreover, when the equations derived from the three-state model were fitted to the data corresponding to all variants (including the wild-type protein)—taking into account shared values of k_IN_, *K*_IU_ and denaturant dependences for all mutants, and different *k*_NI_ rate constants—fittings were still rather acceptable (Table S1 and Figure S1).

**Figure 7:**
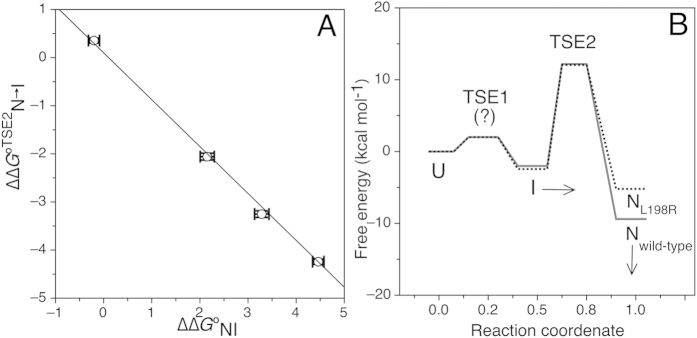
Structure-induced rate-equilibrium free energy relationships involving N and I states. (**A**) The plot shows the correlation between ΔΔ*G*°^TSE2^_N → I_ and ΔΔ*G*°_NI_ eq, the differences in free energy for the activation barrier of the reaction N to I through TSE2 and the free energy of equilibrium between states N and I, respectively. The black line represents a linear regression to the data with a slope = −0.995 ± 0.038. (**B**) Schema of the reaction coordinate proposed in this paper, where U, I, N, are the unfolded, the high-energy intermediate and the native states, respectively. TSE1 and TSE2 are the transition state ensembles between U and I, and between I and N, respectively. For clarity only data corresponding to wild-type and mutant L198R are shown. Arrows show the effect of NaCl on I (compaction) and N (stabilization), whereas (?) indicates lack of information on TSE1 barrier. 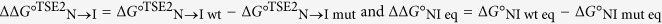.

To evaluate the involvement of the CTR on the energetics of the TSE2, we studied the structure-induced rate-equilibrium free energy relationships. The analysis of our experimental results, in terms of the correlation between ΔΔ*G*°_N → TSE_ and ΔΔ*G*°_NI_ (Fig. 7A), reveals that the CTR does not make a significant contribution to the energetics of the TSE2, relative to its contribution to the N state; this suggests that the CTR is most likely unfolded in TSE2 (see also φ values, in Table 1).

These results indicate that the effect of mutations studied in this work only alter the stability of the native state ensemble and that they are in agreement with the general superimposition of the coefficients k_obs_ observed on the left arms (refolding) in the chevron plots for all variants (Figs 6 and 7B, Tables 1 and 2).

### Structure-based model simulations suggest emerging complexity in the hFXN folding energy landscape

All-atom empirical force fields with explicit solvent can elucidate our understanding of short–time dynamics with high energetic and structural resolution. On the other hand, structure–based models utilize the funneled energy landscape theory of protein folding to study long timescale dynamics^46^,^47^. The native–centric GO model simulations have been massively used to successfully describe folding mechanisms^48^,^49^,^50^. The fact that native structure acquisition has been proven to depend strongly on the protein topology supports the usage of these methods^46^. Given that non-native contacts are not allowed, it follows that non–native structures with non–native contacts are disfavored. The only non–native conformations that can be formed are the ones that appear as a consequence of the balance between enthalpy and entropy (e.g., an entropy–favored configuration)^51^.

**Figure 8:**
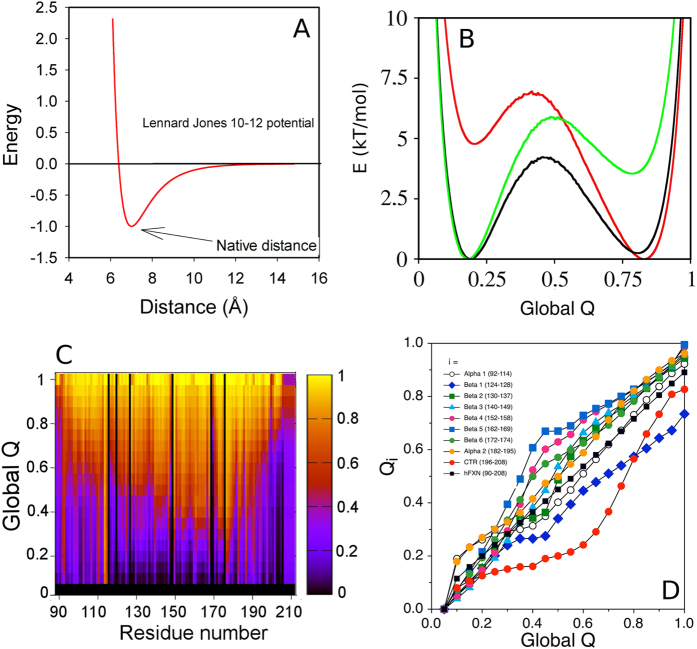
Structure based models: Global Q as the reaction coordinate. (**A**) Non-bonded Lennard-Jones potential for native contacts. (**B**) Free energy profile as a function of global Q at the melting temperature (T_m_, black), below T_m_ (red) and above T_m_ (green). (**C**) Folded state per residue at different global Q values as the likelihood of finding a single residue folded at different positions in the free energy landscape as a function of global Q. (**D**) Folding state per secondary structure elements as a function of global Q. The average Q per residue is shown in filled black circles.

Particularly, the simulation stated in this manuscript is based on the native topology of human frataxin extracted from its X-ray structure (PDB ID 1EKG). Each residue was represented by a sphere centered in the alpha carbon of the respective amino acid residue, as described under “Materials and Methods”. The main concept in this type of simulation is that the only non–bonded contacts that result favorable to the potential energy of the protein are the ones that are defined from the native structure. In this case, these contacts are described by a 10–12 Lennard-Jones potential with a minimum at the native distance; the depth of this well is the same for all native contacts. Residues that are more than three residues away are described by a repulsion term that increases steeply as they approach at a distance below 4A (Fig. 8A). As hFXN topology is well represented in the Protein Data Bank and given that the effect of CTR truncation on protein stability has been previously reported, it was interesting to assess at microscopic level the consequences of altering CTR contacts with the highest contact order.

It has been previously proven that native contacts (Q) are a global reaction coordinate that well represents the folding state of protein-folding mechanisms^52^. Simulations at constant temperatures, following native contacts (Q), make possible the study of the hFXN folding mechanism^20^. Even though the acquisition of structure is strongly biased to the native state ensemble, topological frustration can be studied^51^.

**Figure 9:**
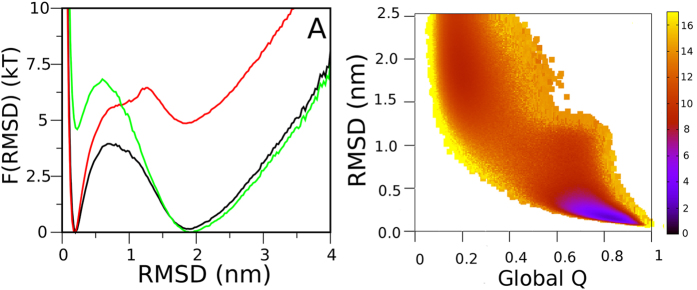
Structure based models: RMSD as the reaction coordinate. (**A**) Free energy profile as a function of RMSD at the melting temperature (T_m_, black), below T_m_ (red) and above T_m_ (green). (**B**) Free energy profile as a function of RMSD and global Q below the T_m_. The color scale represents the free energy (kT mol^**−**1^) calculated as described in Materials and Methods.

Structure-based models followed by native contacts formation yield the free energy landscape shown in Fig. 8B. It can be seen that only two basins are defined: one corresponding to high fraction of native contacts (Q_N_ ~ 0.78) and the other to low fraction of native contacts (Q_U_ ~ 0.17). This suggests a single step reaction. However, Fig. 8C,D show that at Q_i_ values between 0.6 and 0.8, the native contacts involving CTR are not formed. This evidence indicates that in the native basin (defined by Q_global_ as the reaction coordinate), conformations where CTR is folded coexist with conformations where this region is unfolded. Exploration of the root-mean-square deviation of atomic positions (RMSD) as a second reaction coordinate shows that at RMSD values between 0.9 nm and 1.1 nm there is a subtle basin below the melting temperature (Fig. 9A). In Fig. 9B, which shows the free energy profile taking into account both reaction coordinates (RMSD and Q) below the melting temperature, an intermediate ensemble is clearly evidenced. The corresponding reaction coordinate values (Q, RMSD in nm) for each ensemble are: N ~ (0.77, 0.2), I ~ (0.65, 1.0), U ~ (0.17, 2.0), TSE1 ~ (0.43, 1.2) and TSE2 ~ (0.69, 0.7). Bench work showed that a partially folded state (I state) is observed at low urea concentrations in kinetic experiments. As this structure-based model captures the topological component of the structure without taking into account protein electrostatics and other properties that depend on the protein sequence, these simulations may be mimicking the energetics of a condition where charge effects are screened (analogically to 400 mM NaCl experimental conditions). The compaction of I state, calculated from the simulations using Equation 11 and global Q values as a proxy, yielded 80% of the compaction corresponding to the native state. This suggests that I state is almost as compact as TSE2 (87% of the native state compaction), in agreement with the results obtained at high concentrations of NaCl. Furthermore, the simulations show very low phi values for the CTR in the I, TSE1 and TSE2 ensembles, indicating that this region does not significantly contribute to their energetics, in accordance with rapid mixing experiments that firmly suggest that the CTR is unstructured in TSE2.

### The case of the truncated variant hFXN90-195: an extreme destabilization of the native state ensemble without changes in the energetics of TSE2

**Figure 10:**
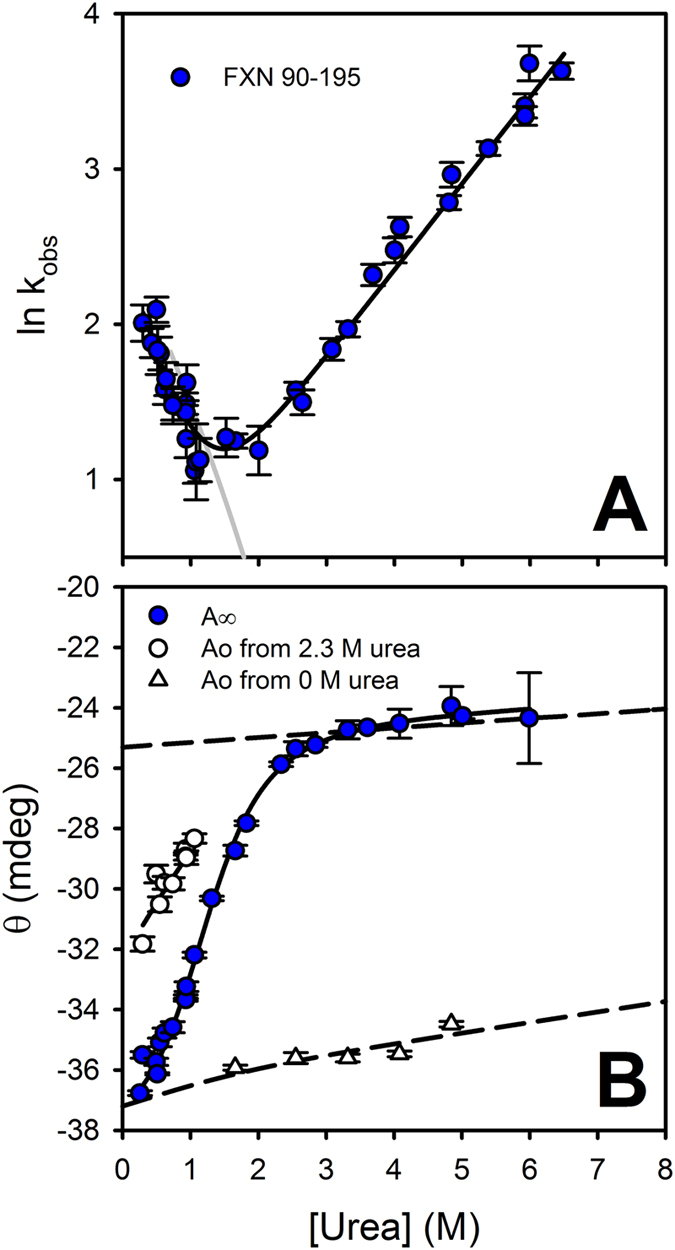
The dependence on urea concentration of k_obs_, *A*_0_ and *A*_∞_ that gave best fit to the experimental data for the truncated variant hFXN90-195. (Panel **A**) is a plot of k_obs_. (Panel **B**) is a plot of *A*_∞_ (signal at t → ∞, filled circles) and *A*_0_ (signal at t = 0), which were obtained from unfolding and refolding experiments (open triangles and circles, respectively). The experiments were carried out at 15 °C and buffer was 20 mM sodium phosphate, 100 mM NaCl, pH 7.0. Prior to the initiation of the refolding reaction, the protein was incubated in a buffer with 2.3 M urea. The continuous lines are the plot of equations derived from the three-state model, (Eqs 2), with the best fitting values of the parameters that gave best fit to k_obs_, *A*_∞_ and *A*_0_ respectively. The dashed lines represent the change in the spectroscopic signal with urea concentration, according to the fractions of native, intermediate and unfolded states at 2.3 M urea. Vertical bars represent the experimental errors.

We previously showed that hFXN90-195 is much destabilized (Table 1) and that the dynamics of its native ensemble are highly altered by comparison with that of wild-type hFXN^19^,^20^. As very high unfolding rates were observed at 25 °C for this variant, we were unable to estimate these parameters with confidence at this temperature. For this reason, we performed the experiments at 15 °C (Fig. 10 and Tables 3 and 4). As for the wild-type protein and point mutants, a single exponential function of time was enough to correctly describe the time traces for the truncated variant. Moreover, as in the case of wild-type hFXN, folding of hFXN90-195 exhibits a burst phase in the refolding curves, likewise indicating the existence of an intermediate in the folding mechanism of the truncated form. However, when the chevron plot is examined a very short refolding branch is evidenced, (Fig. 10A) which comes from a high destabilization of the protein. This occurs with a concomitant large shift of the C_m_ value from 4.9 (wild-type at 15 °C, Fig. 5) to 1.5 M urea (Table 4). In this context, we decided to use the same model as for wild-type hFXN to analyze the folding mechanism of the truncated variant. Remarkably, the difference in the folding free energy obtained from rapid mixing experiments is similar to the one obtained from equilibrium experiments. In addition, the value corresponding to the m_NU_ is the one expected for the truncation of hFXN by 15 residues^53^, and it agrees with the value obtained in equilibrium experiments (Tables 1 and 3). The fact that α_I_ and α_TSE2_ for the truncated and wild-type variants are similar firmly supports the premise that the ensembles visited along the folding routes are shared by both proteins. Although we have no precision in determining *K*_IU_, the k_IN_ constant and m_IU_ dependence (Table 3), we were able to estimate k_NI_ and m_NI_, and concomitantly φ_TSE2_, with confidence (φ_TSE2_ = 1- (ΔΔ*G*°_N->TSE2_/ΔΔ*G*°_NU_)). Interestingly, even in the case of the truncated mutant hFXN90-195, the absence of the complete CTR does not alter the energetics of TSE2 (Table 3), firmly supporting the notion that this stretch is unstructured in this ensemble. It is worthy of note that the analysis of structure-based model simulations of the truncated variant gave rise to the same conclusions, showing that the absence of CTR alters the stability of the native state without altering the transition state ensemble energetics (Figure S2).

As a whole, experimental evidence points to the fact that the presence of the CTR significantly alters the energy gap between native and non-native ensembles, increasing the difference in free energy between N and I state, most likely stabilizing the native state. Thus, the CTR provides hFXN with conformational specificity^54^ and alters the overall topography of the folding landscape.

## Discussion

In this study, we detected the existence of at least one intermediate state in the main folding route of hFXN, suggesting a quite rough folding landscape for this protein. The intermediate is significantly expanded by comparison with the native state (Tables 1 and 2) and the high CD signal (225 nm) associated to it suggests that the intermediate is likely to have a notable helical structure.

It is difficult to say whether the I state is connected with U in a non-cooperative way. The m_UI_ value that we obtained for this transition is low and this fact is in agreement with the presence of a non-compact, partially-folded state. Although models involving a continuum of unfolded states may also account for the rollover behaviour^55^, some important points allow us to distinguish between the I and U states: (i) the I state exhibits much more than a residual secondary structure: ∼60% of the total change in the far-UV CD signal upon folding, most likely, coming from the presence of a high content of the α-helical structure; (ii) the addition of NaCl produces a significant compaction of the I state (a shift from α_urea_ I = 0.44 to 0.71). On the other hand, there is only a slight compaction (∼10%) of the U state, as judged by a decrease in the m_NU_ value (a shift from 1.83 to 1.68 kcal mol^**−**1^ M^**−**1^) at a higher NaCl concentration.

Mutations of the CTR do not alter the activation barrier for folding (I → TSE2), although they highly alter the stability of the native state ensemble (and consequently the barrier for unfolding, N → TSE2). Therefore, the presence of the CTR in hFXN yields an increase in Δ*G*°_NU_ when comparing with the truncated form hFXN90-195, without enhancing the folding rate of the protein. In the same vein, analysis of MDS suggests a three-state folding mechanism for hFXN and indicates a low degree of structure in the CTR in the ensembles TSE1, I and TSE2. This fact could be an enormous advantage for proteins like hFXN that need to be imported into organelles, since they are translocated while being in non-native states^34^.

The fact that residues located in the CTR have very low phi values (Table 1 and Fig. 7) opens the possibility of mutating this stretch to produce hyperstable variants without altering folding and translocation *in vivo*. The significantly more stable mutant hFXN L203C attested this hypothesis. It is noteworthy that Cys at position 203 may establish polar interactions with the side chains of residues His183 and Ser105, and with the backbone oxygen of residue L200 in the native state^4^,^56^,^57^. On the other hand, it will be important to test possible tradeoff relationships between stability and function.

Finally, previous results suggest a correlation between the thermodynamic stability of hFXN and its concentration in cells of heterozygous FRDA patients carrying a GAA expansion and a point mutation^21^,^58^,^59^. Then, we propose that mutations like L203C may be combined with other mutations as K147, which prevents ubiquitin-proteasome-dependent degradation of hFXN^60^,^61^ to produce an increment in hFXN concentration inside the cell, critical for therapeutic approaches.

It was shown for a number of proteins that signal peptides and pro-peptides can alter the population of species in equilibrium and, in some cases, the folding kinetics, making the folding reaction slower than when these regions are absent^35^. Given that in human cells hFXN is produced as a precursor, it is clear that the first 80 residues of the protein may have some relevance in folding/unfolding *in vivo*. The lack of residues 42–80—in a construct of hFXN that has the mitochondrial targeting sequence (residues 1–41) followed by residues 81–210—yields a protein that is correctly processed when expressed in Cos-1 cells. However, the expression level of the mature form inside the mitochondria decreases, and the precursor form accumulates in the cytosol^3^.

Regarding the stability of the native state, the effect of NaCl on hFXN was previously interpreted as a consequence of charge shielding: hFXN has a large cluster of negative-charged residues, which forms an acidic ridge that destabilizes the tertiary structure but is important for hFXN function. The acidic ridge facilitates interaction with IscS (the cysteine desulfurase)^2^ and confers hFXN with iron binding capability^62^,^63^,^64^. In the same fashion, mutation of the highly frustrated acidic residues of yFXN by alanine yields more stable proteins^65^. As mentioned above, Vilanova and coworkers described an intermediate state in equilibrium for yFXN^33^. This intermediate is locally unfolded and exhibits localized energetic frustration that involves acidic side chains. Interestingly, the equilibrium may be shifted to the native-like state by the addition of salt. In this context, it is not surprising that an increment in NaCl concentrations significantly alters the compactness of the intermediate state of hFXN and stabilizes the native state of this protein *in vitro*.

The existence of differences in the folding mechanism among protein homologs is not unusual. On the other hand, folding landscapes seem to be malleable. Even for Trp-cage (a very simple protein) the folding landscape can become extremely complex and significantly altered by simple variations of the charge states of the sequence^66^. In the case of the bovine acyl-CoA binding protein, which exhibits complex equilibrium and kinetic behaviours, it was shown that the structural features of a native-like intermediate are linked to interplay between packing and electrostatics. Furthermore, the intermediate may determine the functionality of this protein, which is characterized by broad substrate specificity^67^. In addition, results in the multistate folder Barstar^68^ indicate that its folding mechanism is the outcome of evolutionary pressure to maintain its binding affinity with Barnase through a large negative electrostatic potential on one face. A single change in the protein sequence (E76K or E80K) at the binding site of Barstar enhances native state stability and alters the Barstar folding mechanism to resemble an unfrustrated two-state-like system. In a context in which the acquisition of the native conformation is robust, all these results point to the high plasticity of the folding mechanism and its sensitivity to mutations.

The whole picture that arises from the analysis of hFXN folding kinetics is substantially more complex than the one recently suggested for the yFXN homolog^26^,^27^. In the case of yFXN, the curvature of the chevron plots was interpreted as a change in the compaction of the transition state ensemble under the assumption of a two-state model with a broad transition barrier. On the other hand, the detection of a burst phase in the hFXN refolding time traces firmly indicates the existence of an intermediate state, thus revealing that the curvature of its chevron plot is a consequence of the relationship between structure and the energetics of the conformational ensembles U, I and N. We think that the frataxin (CyaY) protein family is an excellent model to study how folding and functional landscapes interlink and how functional constraints can govern the folding mechanism.

## Methods

### Expression and Purification of hFXN Variants

Full-length variants (hFXN90-210) wild-type, L198R, L198A, L200A, L203A and L203C were expressed in *Escherichia coli* BL21(DE3) cells and purified from the soluble fractions under native condition, as previously described^19^. On the other hand, variant hFXN90-195 (a truncated form lacking the CTR) was prepared from inclusion bodies, refolded by dialysis, and purified under native conditions, yielding 100 mg of protein per liter of cell culture, with a purity ≥95% (checked by SDS-PAGE). ESI-MS (Thermo Finnigan) was used to confirm the expected masses of the proteins.

In the case of variant L203C, it was not necessary to add a reducing agent since in its absence, free-thiol measurements yielded 1.0 mole of free thiol per mole of protein in the presence of 2–3 M urea (data not shown), and accessibility of Cys203 to the solvent in the native state is very low, as judged by its low reactivity with 5,5′-dithiobis-(2-nitrobenzoic acid). In addition, this variant behaves as a monomer in solution (SEC-FPLC and light scattering measurements), indicating the absence of intermolecular interactions.

### Spectroscopic Characterization of hFXN Variants

Circular dichroism (CD) measurements were carried out with a Jasco J-810 spectropolarimeter. Near-UV and far-UV CD spectra were collected using cells of 1.0 and 0.1 cm path-length, respectively. Data were acquired at a scan speed of 20 nm/min and at least three scans were averaged.

Steady-state intrinsic fluorescence measurements were performed in a Jasco FP-6500 spectrofluorometer operating in the ratio mode. A 0.3 cm path-length cell sealed with a Teflon cap was used. Intrinsic fluorescence of proteins was measured using a protein concentration of 10 μM; excitation wavelength was 295 nm and emission data were collected in the range of 305–450 nm. The spectral slit-widths were set to 3 nm for both monochromators.

Both for CD and fluorescence measurements, proteins were used at a concentration of 10 μM, in a 20 mM Tris-HCl buffer, pH 7.0, containing 100 mM NaCl. Experiments were performed using a thermostated cell holder connected to a circulating water bath set at 25 °C.

### Equilibrium Unfolding Experiments

Isothermal unfolding experiments were carried out incubating hFXN variants for 3–5 h with 0 to 8.0 M urea in a 20 mM Tris-HCl buffer, pH 7.0, containing 100 mM NaCl. Measurements were done at 25 °C and at 15 °C. The process was followed by far-UV CD and tryptophan fluorescence. To determine the thermodynamic parameters, we implemented both a two-state unfolding mechanism, where only native (N) and unfolded (U) conformations exist in equilibrium (U↔N), and a three-state unfolding mechanism, in which an intermediate (I) form exists in equilibrium (U↔I↔N). Data were processed according to Santoro and Bolen^69^.

### Folding Dynamics Studied by Rapid Mixing Experiments

Time courses were carried out with a stopped-flow reaction analyzer (Bio-logic SFM-400) attached to a circular dichroism (CD) detector (spectropolarimeter Jasco 810). In addition to folding and unfolding traces, we carried out experiments where unfolded protein was mixed with urea at high concentrations, and native protein was mixed with buffer solution supplemented with low urea concentrations (unfolding and refolding baselines). CD was measured at 225 nm with a band-pass of 4 nm, using a 0.2 cm path cell. In each experiment, 8000 data points were collected. Between 3 and 6 experimental traces were averaged to evaluate the time course at each urea concentration.

### Data Analysis and Model Selection

One exponential, one exponential plus a straight line, or two exponential functions of time were fitted by nonlinear regression to the average traces.

Regression procedures defined the goodness of fit of a given equation to the experimental data, allowing us to choose the model that best explains the results using a minimal number of parameters by applying the *Akaike Information Criterion* (AIC)^70^. AIC is defined by Equation 5:


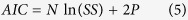


where N is the number of data points, P is the number of parameters plus 1, and SS is the sum of the weighted square residual errors. An equation is considered the best when it gives the lowest value of AIC.

In our case, we found that the best equation according to AIC is a single exponential function of time plus a time-independent term (see the Results section). k_obs_, *A*_0_ and *A*_∞_ values obtained from the best fittings were simultaneously studied in terms of the three-state kinetic/equilibrium model.

Statistical weights were 1 for time courses and the inverse of the standard error for the fitting of k_obs_, *A*_0_ and *A*_∞_. The best fitting values of the parameters were expressed as mean ± standard error.

### Considerations in Folding and Unfolding Experiments

The dependence of each rate constant with denaturant can be expressed as follows (**Equation** 6):


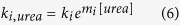


Each signal follows a linear dependence with denaturant concentration (**Equation** 7):


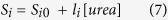


where S_i0_ is the signal at 0 M urea and l_i_ is the dependence of the signal of the state i (U, I or N) with denaturant concentration.

In addition, the relative sensitivities of the intermediate and TSE2 to changes in denaturant concentration were calculated using **Equation** 8 and 9, respectively.


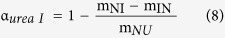



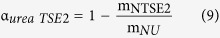


Thus, α_urea (TSE2)_ is the dependence on denaturant concentration of kinetic folding rates I→TSE2 (m_IN_) relative to the difference in free energy of unfolding (m_NU_) whereas α_urea(I)_ is the dependence on denaturant concentration of the difference in free energy between U and I states (m_UI_) relative to m_NU_. The effect of an individual mutation on the stability of TSE2 relative to the reference states (U and N) was measured using **Equation** 10:


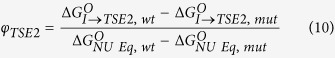


This is a measurement of how native-like is the contribution of this residue to the energetics of the TSE2. When destabilization of N is equal to destabilization of TSE2, then φ_TSE2_ = 1. On the contrary, when the mutation destabilizes N but not TSE2, then φ_TSE2_ = 0.

### Structure-based Simulations

To investigate hFXN folding, structure-based simulations of hFXN were performed^47^,^71^. Each residue is represented by a single bead centred on its alpha carbon position. Adjacent beads are strung together into a polymer chain by means of a potential encoding bond length and angle constraints through harmonic potentials. The secondary structure is encoded in the dihedral angle potential and the non-bonded (native contact) potential. The interaction energy for a given protein conformation and other details are given elsewhere^20^. We used the simulation package GROMACS 4.5.4; the topology, structure, and contact map inputs were calculated using the SMOG server^47^. Several constant-temperature runs were carried out and results were analyzed by the weighted histogram analysis method (WHAM^72^), using Q (fraction of native contacts) as the main reaction coordinate^73^. Folding state per residue is defined as the average number of contacts formed by each residue relative to the total number of contacts formed by that residue in the native state; this local parameter is evaluated at different global fractions of native contacts.

Phi value for a residue, or a group of residues (φ-sim) for a given ensemble, can be calculated using **Equation** 11:


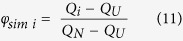


where Q_i_ is the average number of contacts formed by a group of residues in the ensemble I, and Q_U_ and Q_N_ are the average number of contacts formed by these residues in the unfolded and native ensembles, respectively.

## Additional Information

**How to cite this article**: Faraj, S. E. *et al.* Human Frataxin Folds Via an Intermediate State. Role of the C-Terminal Region. *Sci. Rep.* **6**, 20782; doi: 10.1038/srep20782 (2016).

## Supplementary Material

Supplementary Information

## Figures and Tables

**Table 1:** Folding Kinetics and Equilibrium Analysis of hFXN Variants.

Kinetic Experiments (Three-state Model)^a^							Equilibrium Experiments (Two-state Model)^g^		
Variant	bΔ*G*°_NU_(kcal mol^−1^)	cm_eq_ (kcal mol^−1^M^−1^)	dCm _NU_(M)	eφ_TSE2_	fα_I_	fα_TSE2_	Δ*G*°_NU_(kcal mol^−1^)	m_NU_ (kcal mol^−1^M^−1^)	Cm (M)
WT	9.4 ± 0.1	1.91 ± 0.03	4.92 ± 0.01	—	0.44 ± 0.04	0.72 ± 0.01	9.1 ± 0.2	1.83 ± 0.05	4.90 ± 0.04
L198A	6.8 ± 0.2	1.94 ± 0.06	3.51 ± 0.02	−0.03 ± 0.06	0.51 ± 0.07	0.70 ± 0.01	6.6 ± 0.2	1.83 ± 0.05	3.57 ± 0.04
L198R	5.2 ± 0.2	2.02 ± 0.08	2.57 ± 0.02	−0.08 ± 0.03	0.60 ± 0.02	0.76 ± 0.01	4.8 ± 0.2	1.83 ± 0.05	2.62 ± 0.04
L200A	6.0 ± 0.3	1.93 ± 0.10	3.11 ± 0.03	−0.03 ± 0.04	0.52 ± 0.04	0.76 ± 0.01	6.0 ± 0.2	1.83 ± 0.05	3.3 ± 0.04
L203C	10.2 ± 0.1	1.94 ± 0.01	5.26 ± 0.01	0.19 ± 0.16	0.50 ± 0.01	0.76 ± 0.03	9.9 ± 0.3	1.83 ± 0.05	5.37 ± 0.04
90–195	—	—	—	—	—	—	1.6 ± 0.2	1.26 ± 0.013	1.27 ± 0.14

^a^Results from the fitting of a three-state model to the data obtained from kinetic experiments followed by ellipticity at 225 nm, using parameters described in Table 1.

^b^Δ*G*°_NU_ is calculated as Δ*G*°_UI_ + Δ*G*°_IN_.

^c^m_eq_ is calculated as the sum m_eq_= m_IU−_m_IN_+m_NI_.

^d^C_m NU_ is calculated as 0 = Δ*G*°_NU_ + m_eq_ C_m NU_.

^e^φ_TSE2_ is calculated using **Equation** 10.

^f^α_I_ and α_TSE2_ were calculated according to **Equations** 8 and 9, respectively.

^g^From the fitting of a two-state model to the data obtained from equilibrium experiments followed by CD at 222 nm in this study (L200A) and in^19^ for the rest of the mutants.

**Table 2:** Parameters Obtained from the Fitting of Kinetics Experiments.

Kinetic Experiments (Three-state Model)^a^						
Variant	*K*_IU_	*k*_IN_ (s^−1^)	*k*_NI_ (s^−1^)	bm_IU_ (kcal mol^−1^M^−1^)	bm_IN_ (kcal mol^−1^M^−1^)	bm_NI_ (kcal mol^−1^M^−1^)
WT	0.033 ± 0.007	34.6 ± 5.9	(14 ± 1.1) 10^**−**5^	0.83 ± 0.05	−0.54 ± 0.07	0.53 ± 0.01
L198A	0.074 ± 0.030	32.8 ± 7.0	0.0046 ± 0.0004	0.99 ± 0.11	−0.36 ± 0.15	0.59 ± 0.01
L198R	0.016 ± 0.006	20.37 ± 1.61	0.185 ± 0.009	1.21 ± 0.07	−0.32 ± 0.04	0.49 ± 0.01
L200A	0.032 ± 0.017	29.0 ± 4.6	0.0358 ± 0.0029	1.01 ± 0.09	−0.46 ± 0.09	0.46 ± 0.01
L203C	0.012 ± 0.001	26.8 ± 1.8	(79 ± 3.6) 10^**−**6^	0.97 ± 0.02	−0.51 ± 0.02	0.46 ± 0.01

All experiments were performed at 25 °C in buffer 20 mM sodium phosphate, 100 mM NaCl, pH 7.0.

^a^Fitting of equations to the data was performed as indicated in sections Materials and Methods, and Results, and parameters were obtained under rapid equilibrium assumption (between U and I).

^b^Parameters m_IN_ and m_NI_ are the dependences of the rate constants on urea concentration, whereas m_IU_ is the dependence of the equilibrium constant *K*_IU_ on urea concentration.

**Table 3:** Parameters Obtained from the Fitting of Kinetics Experiments.

Kinetic Experiments (Three-state Model)^a^						
Variant	*K*_IU_	*k*_IN_ (s^−1^)	*k*_NI_ (s^−1^)	bm_IU_ (kcal mol^−1^M^−1^)	bm_IN_ (kcal mol^−1^M^−1^)	bm_NI_ (kcal mol^−1^M^−1^)
WT	0.034 ± 0.003	13.4 ± 1.0	(163 ± 4.8) 10^**−**7^	0.80 ± 0.02	−0.57 ± 0.02	0.56 ± 0.01
90–195	0.78 ± 0.97	19 ± 12	1.13 ± 0.05	0.36 ± 0.2	−0.6 ± 0.2	0.31 ± 0.01

All experiments were performed at 15 °C.

^a^Fitting of equations to the data was performed as indicated in sections Materials and Methods, and Results, and parameters were obtained under rapid equilibrium assumption (between U and I).

^b^Parameters m_IN_ and m_NI_ are the dependences of the rate constants on urea concentration, whereas m_IU_ is the dependence of the equilibrium constant *K*_IU_ on urea concentration.

**Table 4:** Folding Kinetics Analysis of hFXN Variants.

Kinetic Experiments (Three-state Model)^a^						
Variant	bΔ*G*°_NU_(kcal mol^−1^M^−1^)	cm_eq_ (kcal mol^−1^M^−1^)	dCm _NU_(M)	eφ_TSE2_	fα_I_	fα_TSE2_
WT	9.86 ± 0.05	1.94 ± 0.01	5.08 ± 0.10	—	0.41 ± 0.01	0.71 ± 0.01
90-195	1.51 ± 0.3	1.15 ± 0.19	1.31 ± 0.33	0.25 ± 0.06	0.40 ± 0.13	0.80 ± 0.03

^a^Results from the fitting of a three-state model to the data obtained from kinetic experiments followed by ellipticity at 225nm, using parameters described in Table 3.

^b^Δ*G*°_NU_ is calculated as Δ*G*°_UI_ + Δ*G*°_IN_.

^c^m_eq_ is calculated as the sum m_eq_ = m_IU_−m_IN_ + m_NI_.

^d^C_m NU_ is calculated as 0 = Δ*G*°_NU_ + m_eq_ C_m NU_.

^e^In this case φ_TSE2_ =  1− (RTln(k_NI wt_/k_NI 90-195_))/(Δ*G*°_NU eq wt_ − Δ*G*°_NU eq 90–195_) because k_IN_ was determined with much less confidence than k_NI_.

^f^α_I_ and αTSE2 were calculated according to **Equations** 8 and 9, respectively.
