# Supplementary material for: Human Frataxin Folds Via an Intermediate State. Role of the C-Terminal Region
Source: Sci Rep. 2016 Feb 9;6:20782. doi: 10.1038/srep20782 (PMC4746760; doi:10.1038/srep20782)
Supplement: Supplementary Information [file srep20782-s1.pdf]

Human Frataxin Folds Via an Intermediate State. Role of C-Terminal Region

Supplementary Information

Santiago E. Faraj, Rodolfo M. González Lebrero, Ernesto A. Roman* and Javier Santos*

Instituto de Química y Físico-Química Biológicas, Universidad de Buenos Aires, Junín 956, 1113AAD, Buenos Aires, Argentina

*Correspondence should be addressed to: ernest.roman@gmail.com and javiersantosw@gmail.com

Tel: +54 11 49648290, ext. 108. Fax: +54 11 49625457 ext. 118

Keywords: CyaY protein family, folding mechanism, iron binding protein, thermodynamic stability, kinetic stability, transition state ensemble, intermediate state, folding kinetics, free energy landscape

Table S1. Parameters obtained from the global fitting of rapid mixing experiments.

**Kinetic Experiments (Three-state Model)[a]**			
**Variant**	k_{NI} (s^{-1})	[b]ΔG^o_{NU} (kcal mol^{-1})	[c]Cm $_{NU}$ (M)
**WT**	$2.0\ 10^{-4}$±$6.2\ 10^{-6}$	8.99±0.04	4.86±0.1
**L198A**	0.0090±0.0003	6.73±0.04	3.64±0.05
**L198R**	0.177±0.004	4.97±0.03	2.69±0.05
**L200A**	0.0025±0.0008	6.12±0.04	3.31±0.07
**L203C**	$5.07\ 10^{-5}$±$1.6\ 10^{-6}$	9.80±0.03	5.30±0.09

[a] Fitting of equations to the data was performed as indicated in sections Materials and Methods, and Results, and parameters were obtained under rapid equilibrium assumption (between U and I). The equations derived from the three-state model were fitted to the data corresponding to all variants (including the wild-type protein) considering common values for k_{IN} (24.9±1.1 s^{-1}), K_{IU} (0.032±0.002) and denaturant dependences m_{NI}, m_{IN} and m_{IU} (0.497±0.003, 0.40±0.02 and 0.94±0.02 M^{-1}, respectively), allowing different k_{NI} rate constants for each mutant. In addition, m_{eq} is calculated as the sum $m_{eq} = m_{IU} - m_{IN} + m_{NI} = 1.85 \pm 0.04$ kcal $mol^{-1}M^{-1}$

[b] ΔG^o_{NU} is calculated as $\Delta G^o_{UI} + \Delta G^o_{IN}$

[d] $C_{m\,NU}$ is calculated as $0 = \Delta G^o_{NU} + m_{eq} C_{m\,NU}$

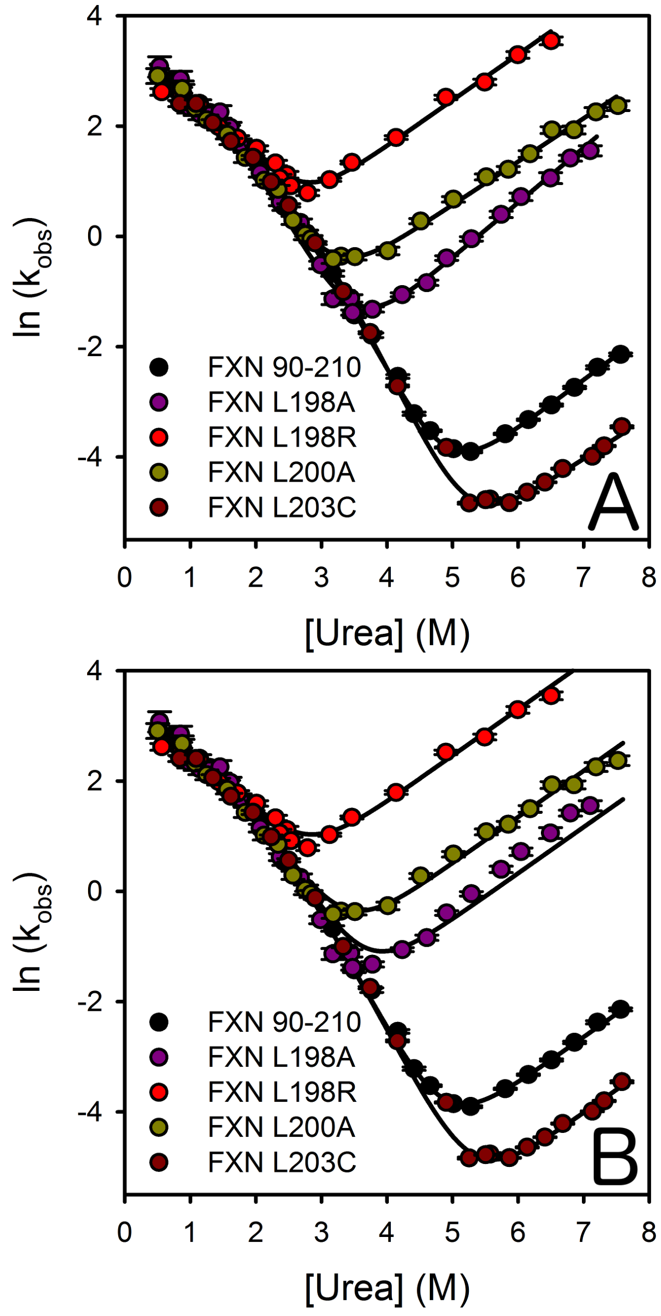

Figure S1. Chevron Plots Corresponding to Wild-type hFXN, L198A, L198R, L200A and L203C. The same data and fittings (three-state model equations) shown in Figure 6 are exhibited in a panel **(A)**. On the other hand, in panel **(B)** the fitting of equations to the data was performed considering shared values for k_{IN} (24.9±1.1 s^{-1}), K_{IU} (0.032±0.002) and denaturant dependences m_{NI}, m_{IN} and m_{IU} (0.497±0.003, 0.40±0.02 and 0.94±0.02 M^{-1}, respectively) among all mutants, and different k_{NI} rate constants for each variant (**Table S1**).

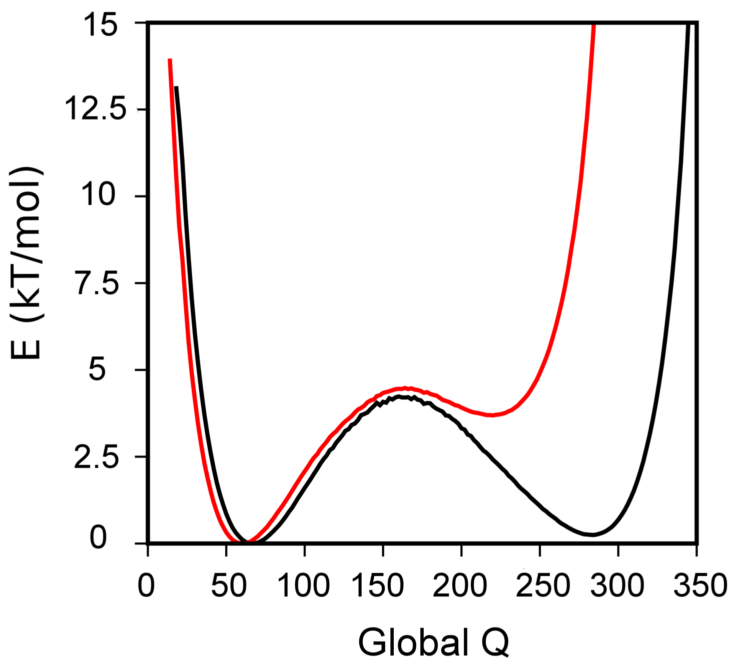

Figure S2. Free energy landscape of hFXN90-210 and hFXN90-195 as a function of the fraction of contacts formed in the native state (Q). Black line shows two basins for the hFXN90-210: the unfolded ensemble is located at a Q of ~0.17, whereas the native ensemble is located at Q ~0.8. Red lines represent the free energy landscape at the same temperature for the truncated hFXN90-195. More importantly, the native state is destabilized; the transition state ensemble and the unfolded states, however, are unperturbed. By using this reaction coordinate, the intermediate state is not detected.
